# Synergistic Effect of *Bacillus subtilis* B3 and β-Glucanase on Solid-State Fermentation of Sunflower Meal

**DOI:** 10.3390/biotech14040092

**Published:** 2025-11-18

**Authors:** Shuqi Chen, Haoran Shi, Peng Zhao, Zengqiang Ma, Xiaolong Li, Xiangyu Wang, Feiyan Xue

**Affiliations:** 1 Key Laboratory for Northern Urban Agriculture of Ministry of Agriculture and Rural Affairs, College of Bioscience and Resources Environment, Beijing University of Agriculture, Beijing 102206, China; 2 Nutrition and Health Research Institute, COFCO Corporation, Beijing 102209, China

**Keywords:** sunflower meal, solid-state fermentation, *Bacillus subtilis*, β-glucanase

## Abstract

Background: Sunflower meal (SFM), a promising feed material, is constrained by its high content of crude fiber (CF) and chlorogenic acid (CGA). Methods: This study utilized a synergistic solid-state fermentation process involving the *Bacillus subtilis* strain B3 and the enzyme β-glucanase to enhance SFM’s application potential. Results: The synergistic treatment notably reduced CF by 12.7% and CGA by 99.77%, while simultaneously increasing acid-soluble protein and reducing sugar by 111.3% and 283.1%, respectively. Positive impacts on its physical structure, characterized by a looser network with visible pores, and on its microbial community, evidenced by an enriched abundance of fungal species such as *Cyberlindnera* and *Aspergillus*, were also observed. In vitro assays indicated improved digestibility of dry matter, neutral detergent fiber, and crude protein, along with a non-significant reduction in methane production. Conclusions: These results demonstrate that microbial-enzymatic synergy effectively enhances SFM’s nutritional profile.

## 1. Introduction

Sunflower meal/cake (SFM), an agricultural by-product, is relatively abundant, inexpensive, and considered a sustainable protein source [1,2]. Typically, SFM consists of more than 25% crude protein (CP), of which 89–99% is true protein, highly beneficial for the proper growth and development of animals [3]. However, SFM also contains anti-nutritional components such as crude fiber (CF) and chlorogenic acid (CGA)—more than 20% and 1% in SFM, respectively, compared to soybean meal. This diminishes its nutritional value and limits its inclusion levels in feed [4,5].

The use of multi-enzymes that target specific substrates in SFM means high cost involved but can potentially allow an increase in its inclusion levels, reducing the deleterious anti-nutritional effects of non-starch polysaccharides. Mbukwane et al. [4] demonstrated that the inclusion rate could be increased to 13.5% when supplemented with xylanase, amylase, and protease. Njeri et al. [6] also examined the effects of pretreating SFM with fiber degrading enzymes (cellulase, β-glucanase, β-mannanase, and pectinase) on components’ solubilization and utilization in broiler chickens. Simović et al. [7] highlighted the significant potential of SFM for the generation of valuable prebiotic compounds through enzyme-aided fractionation. The single enzyme β-glucanase has also been demonstrated to enhance the health of broilers fed wheat-based diets [8], yet no relevant reports on SFM are available.

A cost effective method, solid-state fermentation (SSF), where microorganisms use agro-industrial residues to break down indigestible polymers, such as cellulose and hemicellulose, into simpler sugars, while the following breakdown of indigestible cell wall material releases digestible nutrients, provides potential to increase the dietary inclusion levels [9,10]. The current reports indicated that SSF was usually effective for SFM when the crude fiber content is less than 25%. For example, the initial crude fiber content of 21–22% in SFM was decreased significantly at 72 h of SSF by *Saccharomyces cerevisiae* or *Bacillus subtilis* [3,11,12].

Bacterial enzyme cooperative fermentation, leveraging the synergistic effects of microorganisms and enzyme preparations in the degradation of macromolecular substances, has been demonstrated to enhance nutrient absorption and reduce anti-nutritional factors in feed [13,14]. To our knowledge, there are few studies on the combined effects of microbial strains and enzymes on the SSF of SFM, especially when the crude fiber content is high.

In this study, we evaluated the impact of SSF with the addition of a bacterial strain (*Bacillus subtilis*) and an enzyme (β-glucanase) on the chemical composition, physical structure, microbial community, and in vitro digestibility of SFM with a high crude fiber content, up to 30%.

## 2. Materials and Methods

### 2.1. Materials

SFM, containing CF at 31 ± 1%, CP at 25 ± 1%, CGA at 1.0 ± 0.1%, and crude ash (CA) at 5.6 ± 0.6%, was the de-oiled cake meal provided by COFCO Corporation (Beijing, China). The strain *Bacillus subtilis* B3 (strain B3) was isolated from farmland in Beijing, adapted for growth in SFM, and stored at the China General Microbiological Culture Collection Center (CGMCC) under the registration number 34,578. The enzyme β-glucanase (catalog number: XW18000), belonging to EC 3.2.1.6 and showing high activity at the optimal conditions of 50–60 °C and pH4.0–5.5, was purchased from SUNSON Industry Group Co., Ltd. (Beijing, China). The rumen fluid for the in vitro digestibility test was collected from lactating Holstein cows at the Beijing Dairy Center’s breeding farm. The chemicals used in this study were of analytical grade and were purchased from Sinopharm Group Co., Ltd. in Beijing, China.

### 2.2. Solid-State Fermentation of Sunflower Meal

The de-oiled sunflower cake was ground and passed through a 40-mesh sieve to obtain the substrate of SFM. Strain B3 was cultured in Luria–Bertani medium (10 g/L NaCl, 10 g/L tryptone, and 5 g/L yeast extract) for 18–24 h at 37 °C and 150 rpm until the optical density at 600 nm (OD_600_) reached 0.4–0.6. The SSF system was a proportioned mixture with a material-to-water ratio of 1:1 and was conducted within a breathable bag for 72 h at 37 °C. Four treatments were conducted. In the SFM(CK) group, the composition included only SFM and sterile water. For the SFM-B group, 10^6^ to 10^7^ CFU/g of strain B3 was inoculated. In the SFM-E group, about 90 U/g of β-glucanase was added. For the SFM-(B+E) group, an inoculation of 10^6^ to 10^7^ CFU/g of strain B3 was combined with the addition of approximately 90 U/g of β-glucanase. The fermented products, the contents in breathing bag, were dried for further study.

### 2.3. Chemical Analysis

Samples of different groups were dried, ground, and then passed through a 40-mesh sieve. Subsequently, they were submitted for analysis of CF, acid detergent fiber (ADF), neutral detergent fiber (NDF), CP, acid-soluble protein (ASP), reducing sugar (RS), CA, CGA and pH value. Referring to the method as described by Eide et al. [5] and Njeri et al. [6], the content of CF, ADF and NDF were analyzed using a fully automatic fiber analyzer (ANKOM 2000i, Ankom Technology, New York, NY, USA), and the content of CP and ASP (trichloroacetic acid-soluble material) were analyzed with a fully automatic nitrogen analyzer (Kjeltec™ 8400, Foss, Hillerød, Denmark). The method used for the determination of ASP was as described in the Chinese standard (GB/T 22492-2008) [15]. The RS content was determined using the 3,5-dinitrosalicylic acid (DNS) assay method, calculated from the glucose standard curve. The CA content was determined by assessing the amount of minerals and other inorganic substances left after burning 5 g in a muffle furnace at 550 °C, as specified in the Chinese standard (GB/T 6438-2025) [16]. The content of CGA was determined according to the method described by Sergio et al. [17]. The pH value was measured using a pH meter after a 1:3 (*w*/*v*) ratio of SFM was mixed with water.

### 2.4. Physical Analysis

Samples from various groups were assessed for their color and structure through visual observation. They were also photographed and analyzed using ImageJ. JS (https://ij.imjoy.io, accessed on 10 January 2025) to evaluate the color and particle uniformity. The samples were washed with sterile water, filtered through a 20 μm membrane (NY2004700, Millipore, MA, USA), dried at 50 °C, and further analyzed using scanning electron microscopy (SEM) and Fourier transform infrared spectroscopy (FTIR). A scanning electron microscope (5136 SB, TESCAN, Brno, Czech Republic) was employed to observe the surface morphology of the samples at various magnifications. A Fourier transform infrared spectrometer (Agilent Cary 630, Agilent, CA, USA) was utilized to analyze the chemical groups present in the solid samples. The spectra were recorded in the range of 4000 to 500 cm^−1^, with a scanning resolution of 4 cm^−1^. Before collecting data, background signals were measured and used for correction.

### 2.5. Microbial Analysis

Samples of the four treatments, each with three replicates, were sent to the sequencing company (Shanghai Majorbio Bio-Pharm Technology Co., Ltd., Shanghai, China) to detect the microbial community. Using the genomic DNA of each sample as a template, the bacterial 16S rRNA V3-V4 region was amplified with the forward primer 338F (5′-ACTCCTACGGGAGGCAGCAG-3′) and the reverse primer 806R (5′-GGACTACHVGGGTWTCTAAT-3′). Similarly, the fungal internal transcribed spacer ITS1 region was amplified with the forward primer ITS1F (5′-CTTGGTCATTTAGAGGAAGTAA-3′) and the reverse primer ITS2R (5′-GCTGCGTTCTTCATCGATGC-3′). The amplified products were sequenced using the Illumina Nextseq 2000 (provided by Shanghai Majorbio Bio-Pharm Technology Co., Ltd., Shanghai, China). The high-quality sequences were clustered into different operational taxonomic units (OTUs) based on 97% sequence similarity using UPARSE V7.1 software and aligned with the GreenGenes reference gene database. Alpha diversity was assessed using the Kruskal–Wallis H test on both the Shannon index and the Chao index at the OTU level to estimate biodiversity and microbial abundance within a single sample. Beta diversity analysis was conducted to evaluate differences in microbial community composition and succession, thereby assessing the overall diversity across different samples based on their microbial profiles. Multi-group comparisons of the relative abundance at the phylum and genus levels were performed using a heatmap.

### 2.6. In Vitro Digestibility Test

An in vitro digestibility test was conducted at the Beijing Key Laboratory of Dairy Cow Nutrition. Samples were dried at 55 °C for 48 h, ground through a 2 mm sieve, and then precisely weighed to 0.5 g of fermentation substrate into each culture bottle. The experiment was divided into 8 groups, with 6 replicates per group, totaling 48 culture bottles. To investigate the digestibility, rumen fluid was collected from Holstein dairy cows at the Beijing Dairy Cattle Center breeding farm before their morning feeding. The fluid was then strained through four layers of cheesecloth and maintained at a temperature of 39 °C. The in vitro digestibility of NDF, CP and dry matter (DM) of SFM, as well as its methane production, was determined using the method described by Menke and Steingass [18].

### 2.7. Data Analysis and Statistics

The mean and standard deviation values for three replications (with the exception of the in vitro digestibility test, which had six replications) were calculated in this study. Statistical differences between treatments were assessed using one-way ANOVA, followed by Tukey’s post hoc test in SPSS 21.0. Differences were considered significant when the *p*-value was less than 0.05 and extremely significant when the *p*-value was less than 0.01. The graphs were plotted using the software Origin 2021.

## 3. Results

### 3.1. Results of Chemical Analysis

**Figure 1:**
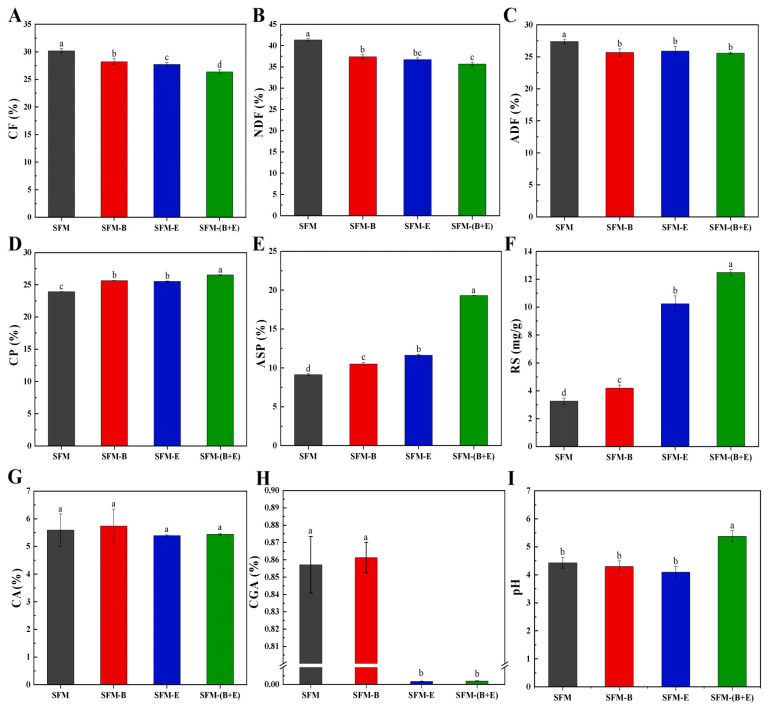
Result of chemical analysis of the raw material (SFM), the fermented products treated by bacterium (SFM-B), enzyme (SFM-E), and synergistic of bacterium and enzyme (SFM-(B+E)). (**A**). Crude fiber (CF). (**B**). Neutral detergent fiber (NDF). (**C**). Acid detergent fiber (ADF). (**D**). Crude protein (CP). (**E**). Acid-soluble protein (ASP). (**F**). Reducing sugar (RS). (**G**). Crude ash (CA). (**H**). Chlorogenic acid (CGA). (**I**). pH value. Different letters indicate significant differences (*p* < 0.05).

As shown in Figure 1, the fermentation processes applied to the SFM resulted in a reduction in CF, NDF, and ADF, and an increase in CP, ASP, and RS. No significant changes were detected in CA, whereas CGA and pH levels exhibited varied changes. After treatment with strain B3, enzyme β-glucanase, and a combination of both strain B3 and β-glucanase, the CF content in SFM was significantly reduced by 6.6%, 8.2%, and 12.7%, respectively (Figure 1A). The content of NDF decreased most notably in SFM-(B+E), with no significant differences observed between SFM-B and SFM-E, or between SFM-E and SFM-(B+E) (Figure 1B). The ADF content also decreased, but no significant differences were observed among the treated groups (Figure 1C).

The results (Figure 1D,E) demonstrated that the CP and ASP contents in SFM-B and SFM-E exhibited a significant difference compared to the control group of SFM. The synergistic treatment involving bacteria and enzymes in the SFM-(B+E) group showed the most significant enhancement, with the CP content increasing from 23.93% to 26.53%, and the ASP content rising from 9.14% to 19.31%. In other words, ASP accounted for 38.19% and 72.79% of the CP content in the control group and the synergistic treatment group, respectively.

The results from the RS content analysis (Figure 1F) demonstrated that fermentation treatments significantly enhanced the availability of glucose in SFM, particularly when β-glucanase was incorporated. The glucose content in the SFM-(B+E) group was increased by 283.1% compared to the SFM group.

The CA content ranged from approximately 5% to 6%, with no significant differences observed among the four groups (Figure 1G).

Notably, the CGA content in the SFM-E and SFM-(B+E) groups was merely 0.01%, indicating a reduction of over 99%. However, in the SFM-B group, it remained as high as in the control group, SFM (Figure 1H).

The treatment with both strain B3 and β-glucanase on the SFM led to an increase in the pH value in the SFM-(B+E) group. However, there was no significant difference in the pH value among the other three groups (Figure 1I).

### 3.2. Results of Physical Analysis

**Figure 2:**
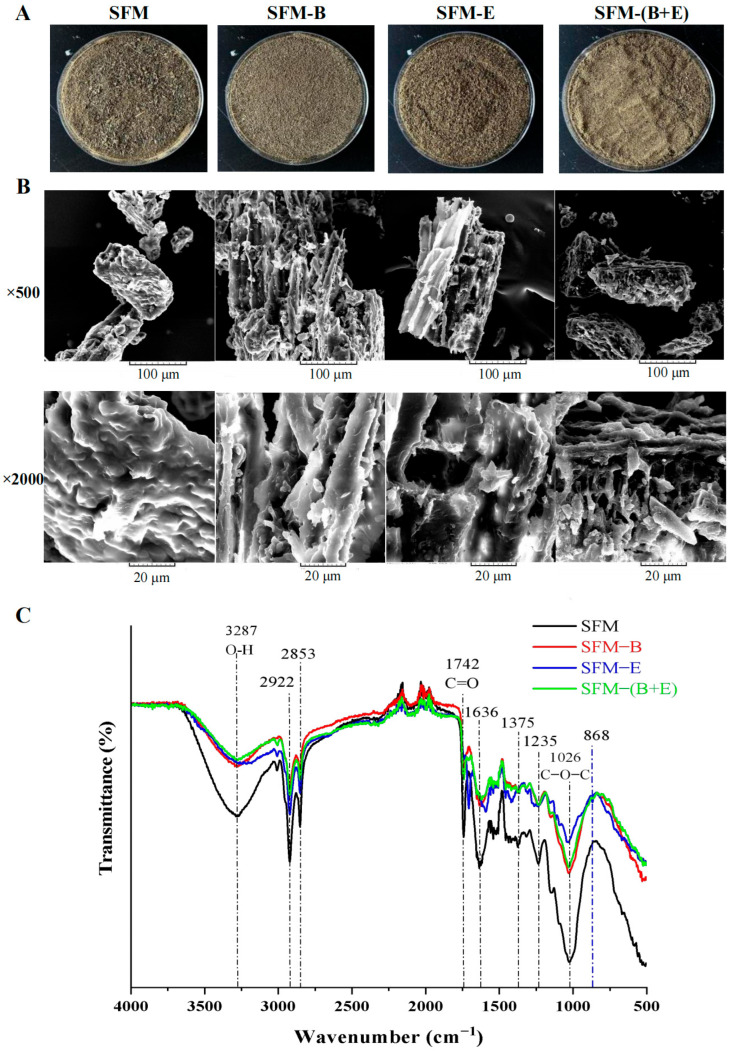
Result of physical analysis of the raw material (SFM), the fermented products treated by bacterium (SFM-B), enzyme (SFM-E), and synergistic of bacterium and enzyme (SFM-(B+E)). (**A**). Appearance images. (**B**). SEM images viewed at 500× and 2000× magnification. (**C**). FTIR spectra.

The structural attributes of the samples were analyzed at both macro and micro scales, as depicted in Figure 2. Compared to the control group, the three treatment groups—namely, the bacterium-treated SFM-B, the enzyme-treated SFM-E, and the synergistic-treated SFM-(B+E)—exhibited lighter color, as well as more uniform texture than the untreated SFM (Figure 2A). The further analysis of quantifying the differences and their significance in the images is demonstrated in Figure S1, where the difference is significant (*p* < 0.05). The morphological characteristics of all four groups were examined using scanning electron microscopy at magnifications of 500× and 2000× (Figure 2B). The surface of SFM particles displayed a relatively smooth texture and a denser lignocellulosic structure. The SFM-B sample’s surface exhibited a looser, lamellar structure. A more porous surface structure was observed in the SFM-E group. The SFM-(B+E) group’s sample displayed networks that were more loosely arranged with diffuse areas and holes, compared to the other three groups. FTIR spectroscopy was applied to analyze the functional groups within solid samples (Figure 2C). In the spectral range studied (4000–500 cm^−1^), all samples displayed telescopic vibrations of O-H at 3287 cm^−1^, C=O at 1742 cm^−1^, and C-O-C at 1026 cm^−1^, Fermi resonance at 2922 cm^−1^ and 2853 cm^−1^, and the intensity of transmittance of SFM was much stronger. The characteristic peak of -OH was markedly diminished following treatment with bacterium and enzyme, particularly after the combination treatment. The characteristic peak of C-O-C was obviously weakened, especially after enzyme treatment. New peaks were also observed between 1600 and 1700 cm^−1^ in the treated groups of SFM.

### 3.3. Microbial Community Analysis

#### 3.3.1. Bacterial Community Analysis

**Figure 3:**
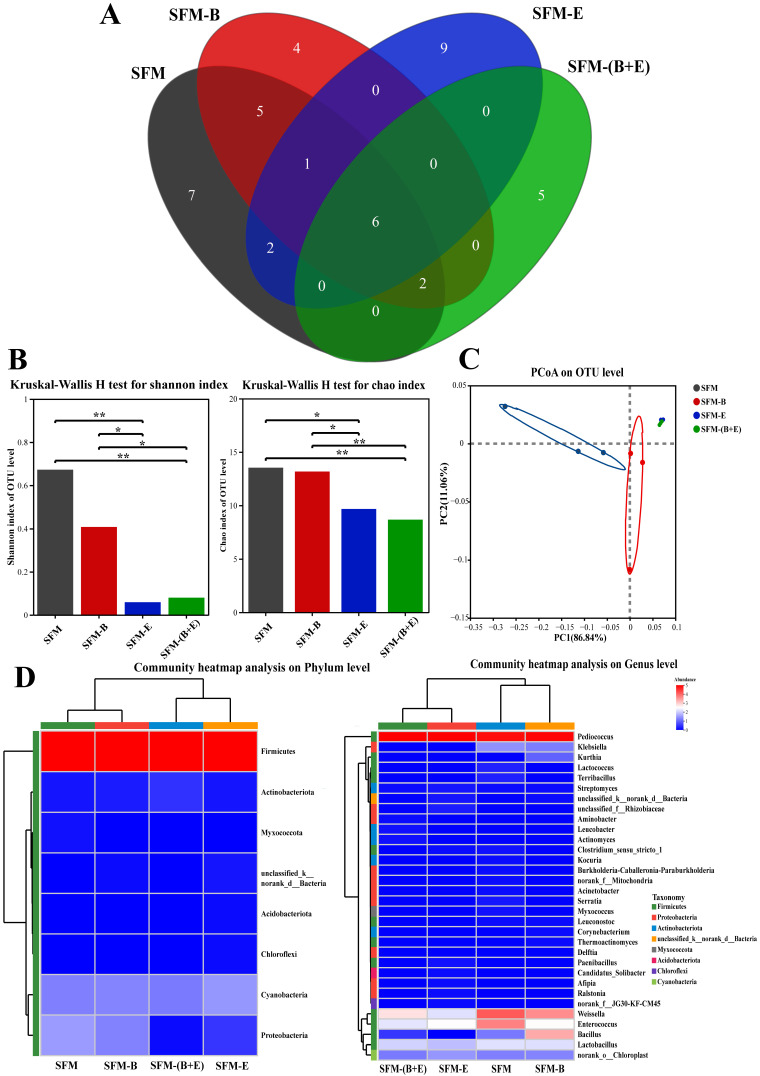
Bacterial community analysis of the raw material (SFM), the fermented products treated by bacterium (SFM-B), enzyme (SFM-E), and synergistic of bacterium and enzyme (SFM-(B+E)). (**A**). Venn diagram analysis based on OTUs. (**B**). Alpha index. (**C**). Beta diversity. (**D**). Community heatmap analysis on phylum and genus levels. * Indicates significant differences at *p* < 0.05. ** Indicates significant differences at *p* < 0.01. In the heatmap, values ranging from 5 to 0 and colors transitioning from red to blue on the scale bar represent the relative abundance of OTUs, from high to low, in the dataset.

The raw reads totaled 826,273, with 816,806 reads retained after quality filtering and optimization for analysis. The quantity, commonalities, and distinctiveness of OTUs across different groups were illustrated in a Venn diagram (Figure 3A). A total of 41 OTUs were identified, with the following distribution among the SFM, SFM-B, SFM-E, and SFM-(B+E) groups: 23, 18, 18, and 12 OTUs, respectively. In the SFM group, there were 7 OTUs of specific bacteria, which accounted for 30.43%. The SFM-B group featured 4 OTUs of specific bacteria, making up 22.22%. The SFM-E group included 9 OTUs of specific bacteria, representing 50.00%. The SFM-(B+E) group had 5 OTUs of specific bacteria, accounting for 38.46%. Collectively, the four groups had 6 OTUs of the same bacteria, representing 8.33%. Additionally, both the SFM and SFM-B groups shared 5 OTUs of the same bacteria, totaling 12.20%. The SFM and SFM-E groups had 2 OTUs of the same bacteria, constituting 4.88%. Across the SFM, SFM-B, and SFM-E groups, there was 1 OTU of the same bacteria, accounting for 1.69%. The SFM, SFM-B, and SFM-(B+E) groups shared 2 OTUs of the same bacteria, which accounted for 3.70%.

As depicted in Figure 3B, the Shannon index indicates that the control group SFM and the bacterial treatment group SFM-B exhibited greater bacterial diversity than the groups SFM-E and SFM-(B+E), which involved enzyme treatment. The difference was significant between groups of SFM and SFM-E (*p* < 0.01), SFM and SFM-(B+E) (*p* < 0.01), SFM-B and SFM-E (*p* < 0.05), SFM-B and SFM-(B+E) (*p* < 0.05). The Chao index for bacterial community abundance in the control group SFM and the bacterial treatment group SFM-B was also higher than in the SFM-E and SFM-(B+E) groups. The difference was significant between groups of SFM and SFM-E (*p* < 0.05), SFM and SFM-(B+E) (*p* < 0.01), SFM-B and SFM-E (*p* < 0.01), SFM-B and SFM-(B+E) (*p* < 0.05).

Figure 3C illustrated the primary component (PC) analysis based on OTUs, wherein PC1 (86.84%) and PC2 (11.06%) demonstrated the differences in bacterial community composition and succession among the four groups. The bacterial treatment group, enzyme treatment group, and the combined bacterial and enzyme treatment group cluster together, located on the right side of the PC1 axis, while the control group clusters on the left side of the PC1 axis. The enzyme treatment group and the combined bacterial and enzyme treatment group cluster together on the upper right side of the PC2 axis, the bacterial treatment group is positioned on the lower right side of the PC2 axis, and the control group is also situated on the lower left side of the PC2 axis.

All effective bacterial sequences were classified from phylum to genus, as depicted in Figure 3D. Based on the assignment results at the phylum level, the primary dominant phylum across the four groups was Firmicutes, which constituted over 99.9% of the total bacterial composition. In the SFM group, the proportions of the first three phyla—Firmicutes, Proteobacteria, and Cyanobacteria—were 99.91%, 0.06%, and 0.03%, respectively. The proportions of all phyla in the SFM-B and SFM-E groups did not differ significantly. In the SFM-(B+E) group, however, the proportion of Firmicutes increased significantly (*p* < 0.05), while that of Proteobacteria decreased significantly (*p* < 0.05). At the genus level, the first four dominant genera in the SFM group were *Pediococcus* (77.21%), *Weissella* (15.88%), *Enterococcus* (6.56%), and *Lactobacillus* (0.22%), respectively. In the SFM-B group, *Enterococcus* decreased significantly (*p* < 0.01) to 0.43% and *Bacillus* increased significantly (*p* < 0.05) from 0.03% to 4.43%. In the SFM-E group, *Pediococcus* significantly increased (*p* < 0.01) to 99.16%, while *Weissella* and *Enterococcus* significantly decreased (*p* < 0.01) to 0.21% and 0.46%, respectively. In the SFM-(B+E) group, *Pediococcus* significantly increased (*p* < 0.05) to 98.76%, and *Weissella* and *Enterococcus* significantly decreased (*p* < 0.01) to 0.79% and 0.24%, respectively.

The results were summarized as follows: first, community structure divergence was observed when different treatments were applied. Next, the combination treatment resulted in an extremely significant decrease in the diversity and abundance of the bacterial community, with the enzyme β-glucanase contributing more than bacterial strain B3. Finally, trends in phylum-level abundance changes for Firmicutes and Proteobacteria were similar when β-glucanase was involved, whereas trends in genus-level abundance changes for *Weissella* and *Enterococcus* differed between SFM-E and SFM-(B+E).

#### 3.3.2. Fungal Community Analysis

**Figure 4:**
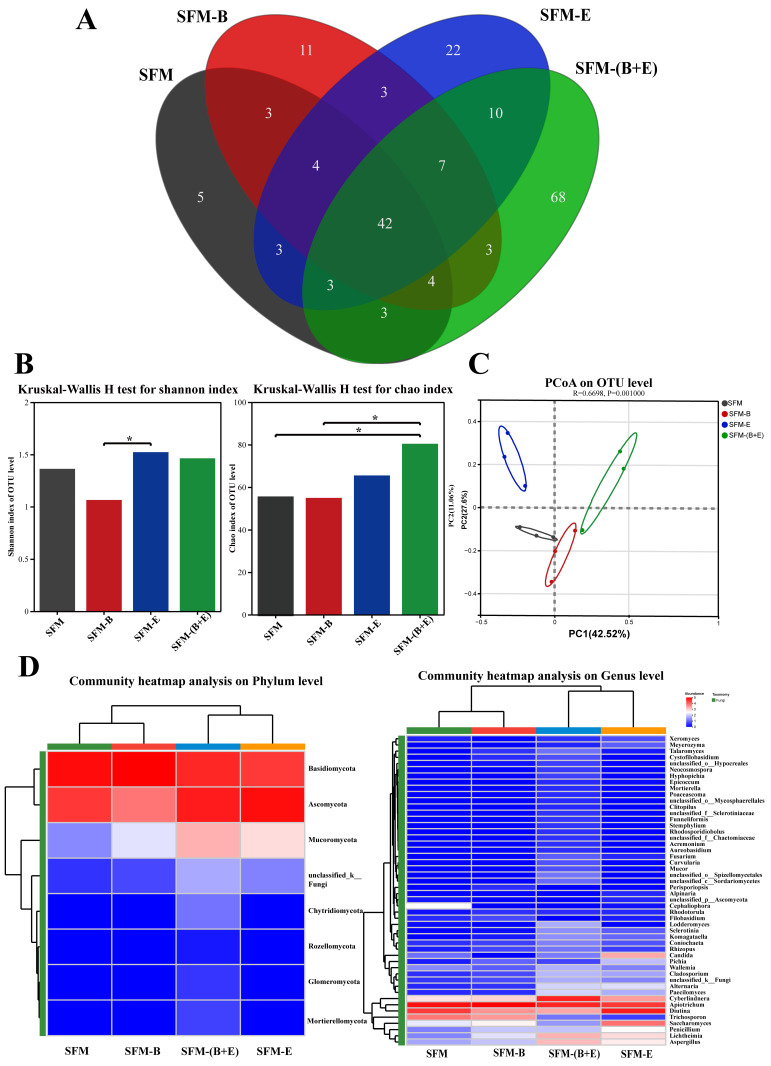
Fungal community analysis of the raw material (SFM), the fermented products treated by bacterium (SFM-B), enzyme (SFM-E), and synergistic of bacterium and enzyme (SFM-(B+E)). (**A**). Venn diagram analysis based on OTUs. (**B**). Alpha index. (**C**). Beta diversity. (**D**). Community heatmap analysis on phylum and genus levels. * Indicates significant differences at *p* < 0.05. In the heatmap, values ranging from 5 to 0 and colors transitioning from red to blue on the scale bar represent the relative abundance of OTUs, from high to low, in the dataset.

The raw reads amounted to 915,327, yielding 870,321 optimized reads for analysis. As shown in the Venn diagram (Figure 4A), a total of 191 OTUs were identified, with 67, 77, 94, and 140 OTUs assigned to the SFM, SFM-B, SFM-E, and SFM-(B+E) groups, respectively. In the SFM group, there were 5 OTUs of specific fungi, which accounted for 7.46%. The SFM-B group had 11 OTUs of specific fungi, making up 14.2%. The SFM-E group contained 22 OTUs of specific fungi, representing 23.40%. The SFM-(B+E) group had 68 OTUs of specific fungi, comprising 48.57%. The overlap between the SFM and SFM-B groups consisted of 53 OTUs of the same fungi, which accounted for 36.81%. The overlap between the SFM and SFM-E groups included 52 OTUs of the same fungi, representing 32.30%. The overlap between the SFM and SFM-(B+E) groups had 52 OTUs of the same fungi, which accounted for 25.12%. The overlap among the three groups of SFM, SFM-B, and SFM-E comprised 46 OTUs of the same fungi, representing 19.33%. The overlap among the three groups of SFM, SFM-B, and SFM-(B+E) included 45 OTUs of the same fungi, which accounted for 22.39%. Finally, the overlap among all four groups consisted of 42 OTUs of the same fungi, representing 11.11%.

Alpha diversity, as indicated by the Shannon and Chao indices, for the four groups was presented in Figure 4B. The Shannon index, which measures fungal community diversity, showed that the SFM-E group had a significantly higher diversity than the SFM-B group (*p* < 0.05). Moreover, there was no significant difference in diversity between the SFM and SFM-(B+E) groups. The Chao index, which reflects fungal community abundance, was significantly greater in the SFM-(B+E) group compared to both the SFM and SFM-B groups (*p* < 0.05).

Beta diversity, as depicted in Figure 4C (the PC analysis based on OTUs, with PC1 contributing 42.52% and PC2 contributing 27.6%), shows the four groups positioned distinctly on the upper right side (SFM-(B+E)), upper left side (SFM-E), lower right side (SFM-E), and lower left side (SFM) of the PC1 and PC2 axes.

The fungal community’s abundance at the phylum and genus levels was characterized and analyzed, with the results presented in Figure 4D. At the phylum level, the first three dominant phyla across the four groups were Basidiomycota, Ascomycota, and Mucoromycota, yet a significant difference was observed (*p* < 0.05). Compared with the control group (SFM), the proportion of Basidiomycota in the SFM-E group was significantly decreased (*p* < 0.05), the proportion of Mucoromycota in the SFM-(B+E) group was significantly increased (*p* < 0.05), and the proportion of Ascomycota in the SFM-E group was extremely significantly increased (*p* < 0.001). At the genus level, the dominant genera in the SFM group, constituting >0.5%, were *Apiotrichum* (62.29%), *Diutina* (26.66%), *Trichosporon* (8.53%), *Cyberlindnera* (0.75%), *Candida* (0.74%), and *Saccharomyces* (0.73%), respectively. In the SFM-B group, *Diutina* experienced a significant decrease (*p* < 0.05) to 6.92%. In the SFM-E group, *Apiotrichum* saw a significant reduction (*p* < 0.05) to 29.1%, while *Diutina* and *Saccharomyces* exhibited significant increases (*p* < 0.05) to 48.88% and 9.45%, respectively. In the SFM-(B+E) group, *Cyberlindnera* showed a significant rise (*p* < 0.01) to 44.26%, *Diutina* decreased significantly (*p* < 0.05) to 3.52%, and *Candida* decreased, though not significantly. *Trichosporon* decreased in the SFM-E and SFM-(B+E) groups, while *Candida* decreased in the SFM-B and SFM-(B+E) groups; however, there was no significant difference observed between the groups.

From the results above, the diversity of fungal OTUs and species abundance in SFM were significantly increased by the combination treatment, particularly for the dominant genus *Cyberlindnera* and the minor genus *Aspergillus*.

### 3.4. Results of In Vitro Digestibility Test

**Figure 5:**
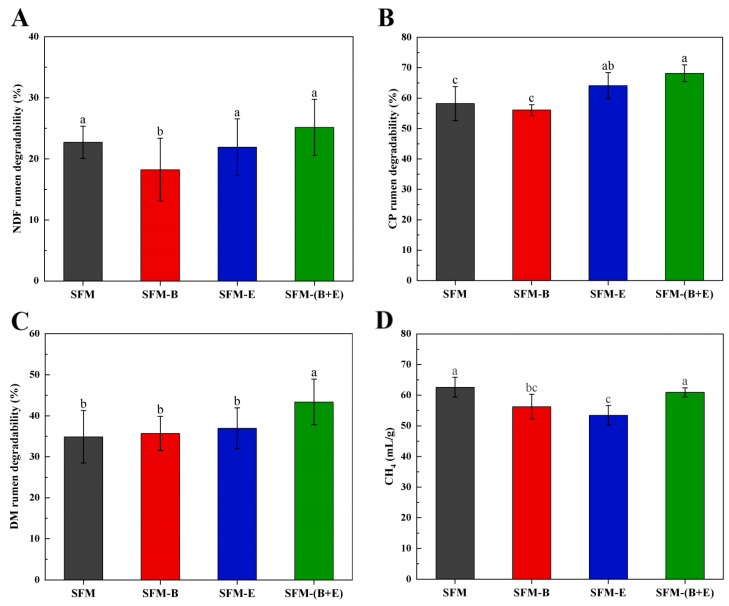
Results of the in vitro digestibility test for the raw material (SFM), as well as for the fermented products treated with bacteria (SFM-B), enzymes (SFM-E), and a combination of both bacteria and enzymes (SFM-(B+E)). The parameters measured include: (**A**). Neutral Detergent Fiber (NDF) rumen degradability, (**B**). Crude Protein (CP) rumen degradability, (**C**). Dry Matter (DM) rumen degradability, and (**D**). Methane (CH_4_) production. Significant differences are indicated by different letters (*p* < 0.05).

The in vitro digestibility test was conducted to assess the rumen degradation rates of NDF, CP, and DM, as well as methane production (Figure 5).

The results indicated that the NDF digestibility of the SFM did not significantly improve with the involvement of β-glucanase. However, a notable inhibition was observed when fermentation was solely conducted with the addition of strain B3, as depicted in Figure 5A.

As depicted in Figure 5B, β-glucanase significantly enhanced the rumen digestibility of CP in SFM, achieving a rate of 64.12%. Notably, when SFM was fermented using both strain B3 and β-glucanase, the rumen degradation rate of CP increased significantly from 58.21% to 68.19%.

The results of Figure 5C showed that the fermentation with both strain B3 and β-glucanase can significantly improve the DM digestion rate of sunflower meal feed, with the DM digestion rate increasing from 42.03% to 49.54%, with a growth rate of 17.86%.

Figure 5D indicates that in vitro rumen gas methane (CH_4_) production was reduced in all treated groups. Compared to the control group utilizing raw material SFM, the SFM-B group, which included only strain B3, and the SFM-E group, which included only β-glucanase, exhibited a significant decrease (*p* < 0.05), with no significant difference between them. In the SFM-(B+E) group, which contained both bacteria and enzyme, the production decreased, but not significantly.

## 4. Discussion

Feeding is a significant cost factor in livestock production, and feed costs contribute 60–80 percent of overall production expenses [19]. Traditionally, soybean meal (SBM) is used as the main protein source in feed. However, the use of imported SBM poses problems both economically and environmentally [1]. SFM could serve as a viable alternative, yet its nutritional quality still requires enhancement. SSF is a common method used in SBM fermentation [20,21,22] and bacteria-enzyme synergy action is more favorable for feed fermentation [13,14,23,24]. Nonetheless, there is limited research on the combined effects of microbial strains and enzymes on the SSF of SFM, especially when the crude fiber content is high. The purpose of this study was to assess the potential for using cost-effective and easily accessible SFM as a substitute for SBM.

### 4.1. Chemical Composition

Chemical composition and nutrient concentration are among the major factors affecting the nutritive value of feeds and ruminal fermentation [25]. SFM contains lower anti-nutritional factors than soybean, but its high content of CF and CGA still limits the use of sunflower meals in feed [12].

Based on the referenced report [12] and the Chinese National Standard (GB 10376-1989) [26], a CF of 28% or above is deemed high and unacceptable. However, the CF content in this study was more than 30%, indicating it must undergo a CF reduction treatment phase. Except for the combination treatment, neither single strain B3 nor the enzyme β-glucanase alone resulted in a degradation rate exceeding 10% (Figure 1A). Therefore, microbial-enzymatic synergy effectively enhances the nutritional profile of SFM for CF.

According to Announcement No. 217 issued by the Ministry of Agriculture and Rural Affairs of the People’s Republic of China in 2019 [27], the recommended addition level of CGA in compound feed is 15–30 mg/kg, which is equivalent to 0.015–0.03%. In Figure 1H, treatments involving β-glucanase significantly reduced the content of CGA in SFM to about 0.018%. This finding is inspiring, as previous studies primarily focused on the removal of CGA using organic solvents, acids, or esterase treatment [17,28,29]. Here, a hypothesis regarding β-glucanase’s ability to degrade CGA and a potential enzymatic mechanism is proposed: the phenolic structure of CGA may interact with the active site of β-glucanase, facilitating enzymatic hydrolysis. This enzymatic approach offers advantages over traditional methods, such as reduced chemical usage and enhanced environmental sustainability. However, further biochemical experiments are required to confirm whether β-glucanase can be used to directly hydrolyze CGA.

ASP was assumed to consist of small molecular peptides and free amino acids, which can be directly absorbed in the animal gut system. In this study, synergistic fermentation involving the *Bacillus subtilis* strain B3 and the enzyme β-glucanase significantly increased the ASP content to as high as 19.31% in SFM (Figure 1E), which was even higher than that of fermented soybean meal (13.48%) as reported by Yao et al. [18], thereby facilitating nutritional improvement. Additionally, the SFM fermented with a combination of strain B3 and β-glucanase resulted in over four times more glucose than other groups (Figure 1F). The limited secretion of endogenous cellulolytic enzymes by a single microorganism during fermentation restricts substrate hydrolysis. The supplementation of β-glucanase mitigates this constraint by introducing exogenous enzymatic activity that synergizes with microbial enzymes, thereby enhancing the efficiency of macromolecular utilization. This synergistic effect aligns with the findings of Lin et al. [30], who observed similar improvements in enzyme-mediated substrate degradation through combined microbial and enzymatic treatments.

### 4.2. Physical Structure

The synergistic fermented product displayed a similar but lighter color, as well as a fluffier and more uniform texture, making it favorable for feed applications. The raw material of SFM has a relatively dense lignocellulosic structure, and which was destroyed into more loosely connected network with diffusion and pores in the SFM-(B+E) group (Figure 2B). To strengthen the experimental basis, comprehensive SEM observations were further conducted, involving images captured from multiple particles, diverse angles, and adjusted magnifications, as presented in Figure S2. These multi-perspective data collectively confirmed that the lignocellulosic structure of the raw material is transformed into a loosely connected network with diffusion and pores. Additionally, a similar phenomenon, where the smooth surface of the raw material is destroyed to form numerous holes, has been observed in the hydrolysis of wheat straw and corn stover by bacteria [31].

The observed changes in the broad peak at 3500–3000 cm^−1^, corresponding to the stretching vibrations of free -O-H, at 1636 cm^−1^, attributed to the aromatic benzene, and at 800–1300 cm^−1^, corresponding to the stretching vibrations of -C-C and -C-O, along with the decrease in the ratio of peak intensities at 2922 cm^−1^ and 2853 cm^−1^, indicated that the raw material of SFM was degraded after the synergistic fermentation, as explained by Ma and Mu [32]. The enzymes, including the endogenous cellulolytic enzymes secreted by the strain B3 and the added β-glucanase, collectively break down the lignocellulosic structure, causing the observed FTIR peak changes: the broad peak at 3500–3000 cm^−1^ shifts due to the exposure of hydroxyl groups after the degradation of cellulose or hemicellulose; the peak at 1636 cm^−1^ weakens due to the degradation of lignin; the peak at 800–1300 cm^−1^ changes as lignocellulosic bonds are cleaved; and the 2922/2853 cm^−1^ peak intensity ratio decreases due to altered aliphatic chain configurations. The peaks between 1600 and 1700 cm^−1^ indicate the amide I band arises principally from the stretching vibration C=O of the peptide group [33]. Therefore, the increase in CP and the extremely significant increase in ASP might contribute to the new peaks between 1600 and 1700 cm^−1^.

### 4.3. Microbial Community

The Shannon index is commonly used to measure species diversity, while Chao1 is usually used to measure species abundance [34]. In this study, the bacterial and fungal communities were analyzed in the four groups following SSF.

The diversity and abundance of bacterial species in SFM were significantly reduced when enzymes were involved, and they were extremely significantly decreased under the combination of strain B3 and β-glucanase (Figure 3B). It is interesting that this decrease parallels the changes in CGA content (Figure 1H) and Proteobacteria abundance (Figure 3D). In the SFM-(B+E) group, the significant reduction in *Weissella* and *Enterococcus* correlates with its significantly higher pH (Figure 1I). This is because these two genera of bacteria prefer acidic conditions. Consequently, the function of β-glucanase creates a substrate and pH alteration, which favors some species while inhibiting others.

There was no significant effect of synergistic fermentation on fungal species diversity in SFM; however, it significantly increased fungal species abundance, particularly for the dominant genus *Cyberlindnera* and the minor genus *Aspergillus* (Figure 4B). This result can be attributed to the synergistic effect of *Bacillus subtilis* B3 and β-glucanase, which caused the degradation of CF and CGA (Figure 1A,H), production of ASP and RS (Figure 1E,F), and an increase in pH (Figure 1I). These changes created a favorable micro-environment for the growth of *Cyberlindnera* and *Aspergillus*. These fungi can further contribute to SFM degradation by producing complementary enzymes to reinforce a positive feedback loop. Consequently, the interplay between enzyme activity, pH, and microbial adaptation is better balanced in the SFM-(B+E) group compared to others. Additionally, the genus *Cyberlindnera* is widely recognized as advantageous for feed purposes. Specifically, the inclusion of *Cyberlindnera jadinii* in dietary regimens has been proven to enhance multiple immune responses and general health in both dairy animals and broiler chickens, as documented by Kidane et al., Zhao et al., and Itani et al. [35,36,37]. *Aspergillus* is one of the most prevalent microorganisms, known for its ability to produce enzymes [18]. In this study, the abundance of *Aspergillus* in the best-performing group, SFM-(B+E), significantly increased (*p* < 0.001) from 0.06% to 1.69% (Figure 4D). Similarly, *Aspergillus niger*-fermented soybean meal and SFM had a positive impact on the growth of *Penaeus vannamei* [38].

Bacteria and fungi were the primary focus of this study. The archaeal community consists almost exclusively of methanogens, and there have been relatively few investigations into archaeal communities related to CH_4_ production in Holstein dairy cows fed with SFM [39]. Therefore, future research will include archaea to fully elucidate microbial dynamics in anaerobic environments, such as in vivo trials.

### 4.4. In Vitro Digestibility and Methane Production

In this study, the synergistic fermentation of strain B3 and β-glucanase significantly improved the nutrient digestibility of CP and DM (*p* < 0.05), increased the fiber digestibility of NDF, though not significantly, and decreased in vitro rumen gas methane (CH_4_) emissions, though not significantly (Figure 5). Referencing the observations and hypothesis of Harsini Shakarami et al. [9], the improved CP and DM digestibility may be due to the loosening of the structural SFM and a reduction in its fiber content caused by fermentation. Greenhouse gas emissions from livestock farming are high, mainly caused by methane emanating from rumen fermentation [19]. Previous studies have indicated that higher fiber content, increased NDF digestibility, and a larger bacterial population, along with lower CP concentrations, can lead to greater gas production [25,40]. Our findings revealed a decrease in fiber content, elevated protein levels (Figure 1D), a reduction in bacterial abundance (Figure 3D), and no significant changes in NDF digestibility (Figure 5A). These results collectively account for the non-significant decrease in CH_4_ production observed in this study.

## 5. Conclusions

In summary, the findings of this study generally suggest that the synergistic fermentation involving the *Bacillus subtilis* strain B3 and the enzyme β-glucanase can modify the chemical composition, physical structure, and microbial community of SFM during solid fermentation, thereby improving its digestibility and nutritional quality. Specifically, there are significant increases in CP, ASP, RS content, and pH value, along with significant reductions in CF, NDF, ADF, and CGA content. Moreover, there is a positive impact on CP and DM digestibility, with no observed effect on methane production, indicating potential applications. The inclusion level of SFM in feed can be notably increased, achieving a proper nutritional balance and performance enhancement. Further studies are required to improve the degradation rate of CF and to establish in vivo trials to assess the performance of fermented SFM and its potential for mitigating greenhouse gas emissions.

## Data Availability

The original contributions presented in this study are included in the article. Further inquiries can be directed to the corresponding author.

## Supplementary Materials

The following supporting information can be downloaded at: https://www.mdpi.com/article/10.3390/biotech14040092/s1, Figure S1: Mean gray value analysis of appearance images for the raw material (SFM), the fermented products treated by bacterium (SFM-B), enzyme (SFM-E), and synergistic of bacterium and enzyme (SFM-(B+E)). The areas analyzed are identical at 20,088 pixels each. Different letters indicate significant differences (*p* < 0.05); Figure S2: SEM images captured from multiple particles, diverse angles, and adjusted magnifications of the raw material (SFM), the fermented products treated by bacterium (SFM-B), enzyme (SFM-E), and synergistic of bacterium and enzyme (SFM-(B+E)).

## Abbreviations

The following abbreviations are used in this manuscript:

SFM	Sunflower meal
SBM	Soybean meal
SSF	Solid-state fermentation
CF	Crude fiber
ADF	Acid detergent fiber
NDF	Neutral detergent fiber
CP	Crude protein
ASP	Acid-soluble protein
RS	Reducing sugar
CA	Crude ash
CGA	Chlorogenic acid
OTU	Operational taxonomic unit
DM	Dry matter
PC	Primary component
