# Supplementary material for: Synergistic Effect of Bacillus subtilis B3 and β-Glucanase on Solid-State Fermentation of Sunflower Meal
Source: BioTech (Basel). 2025 Nov 18;14(4):92. doi: 10.3390/biotech14040092 (PMC12641796; doi:10.3390/biotech14040092)
Supplement: Supplementary file 1 [file biotech-14-00092-s001.zip › biotech-3940208-supplementary.pdf]

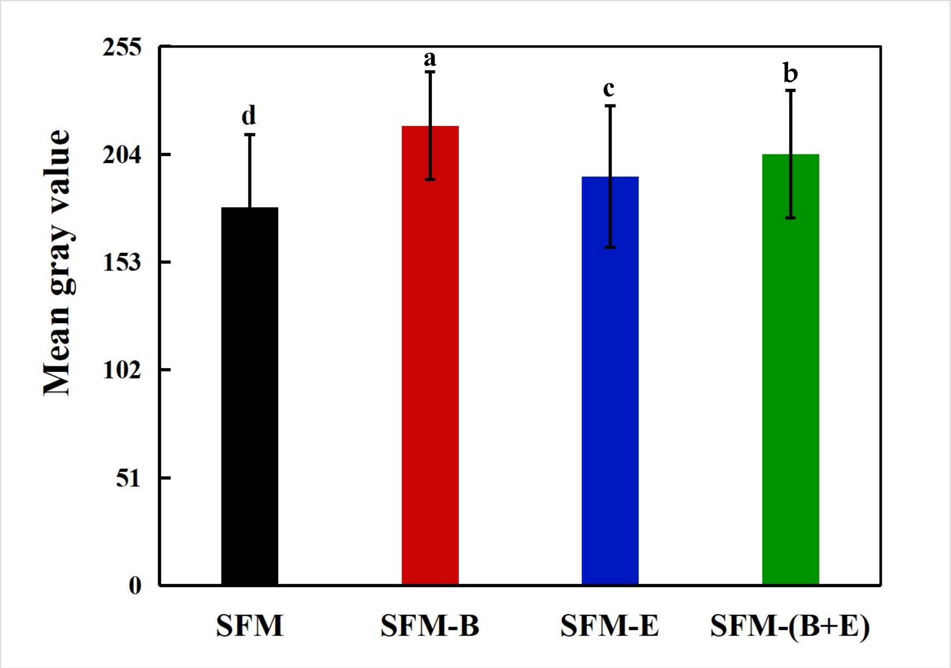

Figure S1. Mean gray value analysis of appearance images for the raw material (SFM), the fermented products treated by bacterium (SFM-B), enzyme (SFM-E), and synergistic of bacterium and enzyme (SFM-(B+E)). The areas analyzed are identical at 20,088 pixels each. Different letters indicate significant differences ($p < 0.05$).

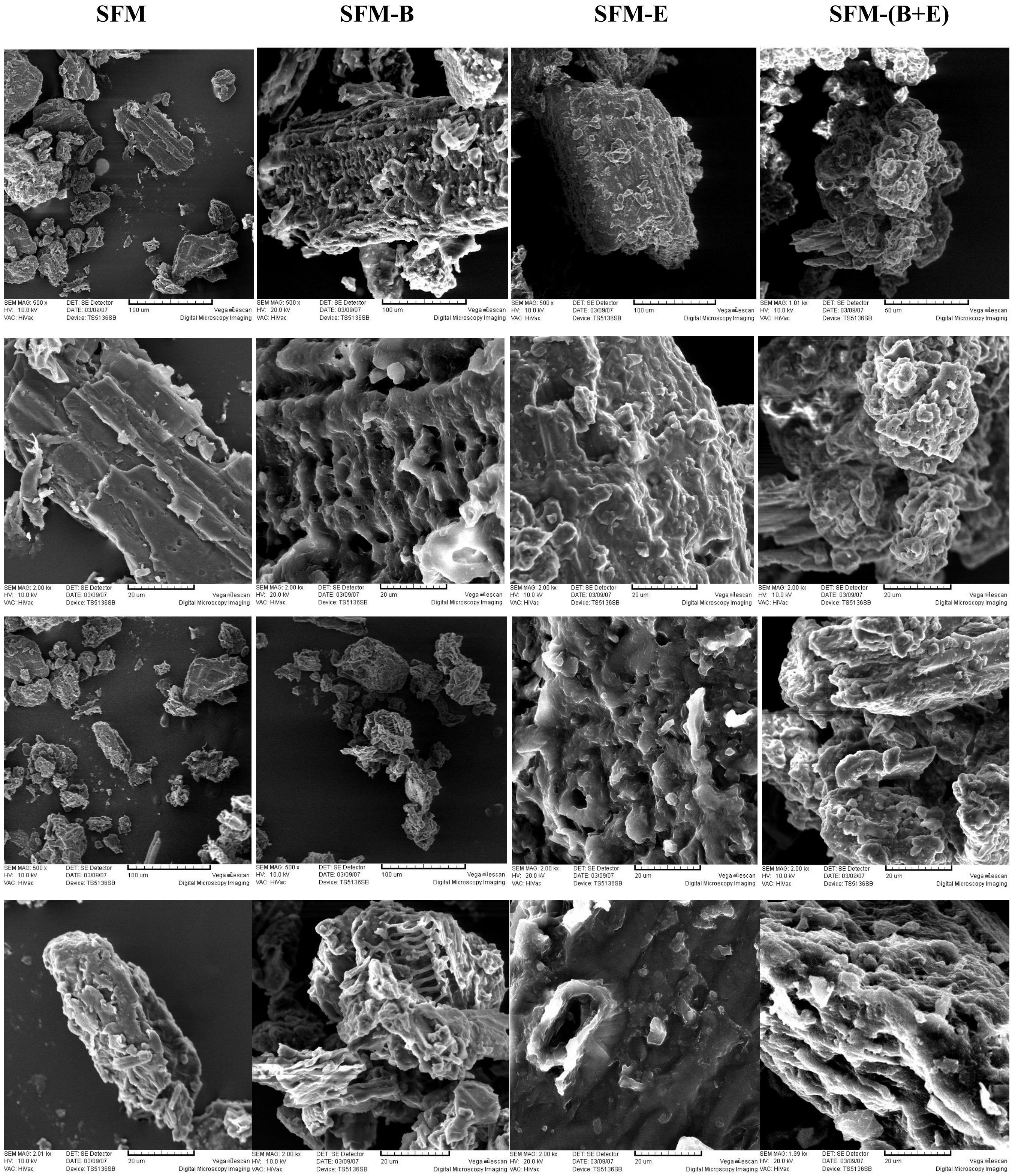

Figure S2. SEM images captured from multiple particles, diverse angles, and adjusted magnifications of the raw material (SFM), the fermented products treated by bacterium (SFM-B), enzyme (SFM-E), and synergistic of bacterium and enzyme (SFM-(B+E)).
